# Human influence on the temporal dynamics and spatial distribution of forest biomass carbon in China

**DOI:** 10.1002/ece3.3188

**Published:** 2017-07-03

**Authors:** Weiwei Liu, Fei Lu, Yunjian Luo, Wenjing Bo, Lingqiao Kong, Lu Zhang, Bojie Liu, Zhiyun Ouyang, Xiaoke Wang

**Affiliations:** ^1^ State Key Laboratory of Urban and Regional Ecology Research Center for Eco‐Environmental Sciences Chinese Academy of Sciences Beijing China; ^2^ University of Chinese Academy of Sciences Beijing China; ^3^ Joint Center for Global Change Studies (JCGCS) Beijing China; ^4^ School of Horticulture and Plant Protection Yangzhou University Yangzhou Jiangsu China

**Keywords:** carbon density, carbon stock, forestation, human activity, population density

## Abstract

Global carbon cycles are impacted by human activity primarily via fossil fuel combustion and forest carbon budget alterations. In this study, the temporal dynamics and spatial distribution of forest biomass carbon (FBC) stock and density in China were analyzed to assess the large‐scale effects of humans on FBC. The results indicated that from 1977 to 2013, the FBC stock increased by 62.9%, from 4,335 to 7,064 Tg C, owing to human‐driven forestation and ecological restoration programs. Because of intensive human impacts, 44.2%–54.6% of the FBC stock was concentrated in four provinces (Heilongjiang, Yunnan, Inner Mongolia, and Sichuan) and the FBC density increased from the densely populated southeastern provinces to the sparsely populated northeastern and western provinces. On a spatial scale, the FBC density was significantly negatively related to population density, and the degree of the dependence of the FBC density on population density has been declining since 1998. This improvement in human–forest relations is related to economic development and programs in China that have promoted forestation and reduced deforestation. These results suggest that human impacts, including forestation, deforestation, population density, and economic development, have played significant roles in determining the temporal and spatial variations of FBC in the anthropogenic era. Moreover, our findings have implications for forest management and improvement of the forest carbon sink in China.

## 1 INTRODUCTION

Forests cover about 30% of the earth's terrestrial land surface and store approximately 33%–46% of the terrestrial organic carbon (Bonan, 2008; Canadell & Raupach, 2008; FAO, 2010). Because of their important role in the conservation of carbon storage and their potential for absorbing CO_2_ from the atmosphere (Vilén, 2015), forests play important roles in mitigation of global climate change (Bonan, 2008; IPCC, 2014). Forest biomass carbon (FBC) exhibits large temporal and spatial variations on both global and national scales (Dixon et al., 1994; Pan et al., 2011; IPCC, 2014). The exploration of FBC spatial–temporal patterns could improve our understanding of the dynamics, processes, and mechanisms of the global carbon cycle (Fang et al., 2014) and promote more effective predictions of atmospheric CO_2_ concentration trends (IPCC, 2001; Coulston, Wear, & Vose, 2015). Previous studies indicated that forests in China have acted as carbon sinks in past decades (Fang et al., 2014; Pan et al., 2011; Piao et al., 2009; Zhang et al., 2013); however, few studies have addressed the spatial variations in the FBC across China (Guo, Hu, Li, Li, & Fang, 2013; Zhang et al., 2013). In addition, the most recent spatial distribution of FBC in China has not been reported.

The FBC stock is an important ecosystem service that is closely related to forest ecosystem quality and is greatly influenced by natural factors and human activities (Ouyang et al., 2016), including climate change, site conditions, fire, insect and disease outbreaks, forestation, harvesting, and conversion of forest to nonlumber uses (Bongers, Chazdon, Poorter, & Peña‐Claros, 2015; Canadell & Raupach, 2008; Hautier et al., 2015; Roberts et al., 2015). Increases in human populations have intensified human activities, which have greatly changed the area and structure of the forest ecosystem (Berenguer et al., 2014; Noble & Dirzo, 1997; Wang, Feng, & Ouyang, 2001) and influenced the FBC stock and its distribution (Keith, Mackey, & Lindenmayer, 2009; Magnani et al., 2007; Tian et al., 2016). Forestation that has been undertaken as part of ecological restoration programs is the most effective method of enhancing the carbon sequestration capacity of forests (Huang, Liu, Shao, & Xu, 2012; Piao et al., 2009). Global forestation has the potential to sequester 60–90 Pg C from 1995 to 2050 (IPCC, 2000). Woodbury, Heath, and Smith (2007) estimated that afforestation in the conterminous United States caused an average of 11 Tg C/year in sequestration on the forest floor from 1990 to 2004. Huang et al. (2012) reported that in China, afforestation caused a net carbon sequestration of 28.15 Tg C/year averagely in forest biomass from 1950 to 2010. Liu and Wu (2014) comprehensively reported the carbon storage and sequestration of the National Key Ecological Restoration Programs, and specific studies have shown that the amount of forest carbon sequestered via the implementation of the Natural Forest Conservation Program, Grain for Green Program, and Three‐North Shelterbelt at provincial or regional scales were 3.6, 2.4, and 1.5 Tg C/year, respectively (Chen, Zhang, Zhang, & Wan, 2009; Liu, Li, Ouyang, Tam, & Chen, 2008; Liu et al., 2014). Proper ecological management, such as the implementation of ecological stewardship projects, might also raise the FBC via ecological reservation, restoration, and construction measures (Liu et al., 2008; Ouyang et al., 2016). Ouyang et al. (2016) indicated that China's national conservation policies contributed significantly to increases in carbon sequestration, which increased by 23.4% between 2000 and 2010.

However, deforestation have accounted for approximately one‐third of the global anthropogenic CO_2_ emissions since 1850 (Rhemtulla, Mladenoff, & Clayton, 2009; IPCC, 2014), and forest degradation caused by human disturbances also represents an important contributor to global carbon emissions (Berenguer et al., 2014). In the Amazonian region, the net effect of deforestation and reforestation caused by human disturbance resulted in an annual net carbon loss of 0.15–0.35 Pg C (Houghton, Gloor, Lloyd, & Potter, 2013). The carbon stock of live vegetation in tropical forests that has been affected by human disturbances (primarily logging, fire, and fragmentation) was 40% lower than that of undisturbed tropical forest in the Brazilian Amazon (Berenguer et al., 2014). In the United States, anthropogenic disturbances (such as land use changes, logging, and land clearing) were the dominant factors influencing the spatial variation of FBC (Goetz et al., 2012). Therefore, human activities might have both positive and negative influences on FBC, and it is essential to clarify the transformation and spatial heterogeneity of these influences to coordinate human–nature relations. Population density is an indicator of human activity that reflects the intensity of human activities, and it may also indicate the influence of human activity on FBC because it represents a significant underlying factor in deforestation (Uusivuori, Lehto, & Palo, 2002), which changes the structure and function of forest ecosystems (Josefsson, Hörnberg, & Östlund, 2009; Wang et al., 2001). Iverson et al. (1994) and Wang et al. (2001) suggested that the carbon density of forest stands decreased as the population density increased in South and South‐East Asia in 1980 and in China from 1984 to 1988. However, it remains to be determined whether the relationship between the FBC density and population density is universal and how this relationship changes with time.

As a nation that is undergoing rapid economic development and social reform, China has experienced sharp increases in population of more than 500 million people over the past four decades (National Bureau of Statistics of China, 2014). During this period, China conducted the world's largest government‐financed programs for forestation (Ouyang et al., 2016). These efforts have increased the forest area by 3 × 10^6^ ha every year since 2000 (FAO, 2010). Because of the increasing population and gradually expanding forest area, the influence of human activities that is indicated by forestation and population density on FBC changes should be assessed. The objective of this work was to evaluate the effects of human activity on FBC dynamics and determine how these effects vary by analyzing the relations of the spatial–temporal patterns of FBC to forest area changes and population density.

## 2 MATERIALS AND METHODS

### 2.1 FBC stock estimation

Compared with the conventional method using wood density and allometric models (Luo et al., 2009; Saatchi et al., 2011), the continuous biomass expansion factor method can be used for all forest types and the parameters are systemic (Fang, Chen, Zhao, & Ci, 2002), and have relatively high precision (Fang et al., 2014). Because the parameters (e.g., wood density, ratio of root and stem, diameter, and tree height) even the model types in the conventional method and allometric models are deeply influenced by site conditions, it is difficult to acquire enough parameters of different geographic location across the vast national area and complicated geography and climate condition in China. Fang et al. (2014) reported that the R‐squared values of the BEF equations used to convert the timber volume to biomass for most forest types were above 0.8, and the estimation error should be less than 3% at the national level. Therefore, we used the continuous biomass expansion factor method to estimate the FBC stock (*C*_stock_, Tg C) by Equation (1).

(1) $$C_{\mathrm{stock}}=C_{c}\sum_{i=1}^{31}\sum_{j=1}^{25}S_{ij}\left(a_{j}V_{ij}+b_{j}\right)$$

where *C*_c_ is the biomass to carbon content coefficient (which was adapted as 0.5) (IPCC, 2003); *S*_*ij*_ and *V*_*ij*_ are the total area (10^3 ^ha) and timber volume per unit area (m^3^/ha), respectively, of forest type *j* (*j* = 1, 2, 3…, 25) for province *i* (*i* = 1, 2, 3 …, 31); and *a*_*j*_ and *b*_*j*_ are constants for specific forest types that are used to convert the stand volume to the stand biomass, respectively, as derived from the literature (Fang et al., 2014; Guo et al., 2013).

The forest area (Data S1) and timber volume (Data S2) were derived from national forest inventories that covered seven periods (1977–1981, 1984–1988, 1989–1993, 1994–1998, 1999–2003, 2004–2008, and 2009–2013) and were compiled by the Chinese Ministry of Forestry (Chinese Ministry of Forestry, 1983, 1989, 1994, 2000, 2005, 2010, 2014). The inventories provided detailed information on the forest area and timber volume according to the age class of the dominant forest type and the origin of the forest stands at the provincial level. The sampling design required a >90% precision in the measurements for the forested area and timber volume from the inventory (Chinese Ministry of Forestry, 2015). Hainan and Chongqing were separated from Guangdong and Sichuan in 1988 and 1997, respectively. Because of lacking data on the forest area and timber volume before the split in the inventory, we did not estimate the FBC of those provinces before split. For this study, “forest” refers only to forest stands with canopy coverage ≥20%, thereby excluding economic forests and bamboo forests. The canopy coverage criterion of forest stands changed from >30% to >20% in China in 1994. To compare the carbon stock on a temporal scale, we corrected the forest areas and estimated the carbon stock before 1994 to >20% under the uniform standards according to the following method used by Fang et al. (2014):

(2) $$S_{0.2}=1.290\;S_{0.3}^{0.995}\left(R^{2}=.996,\;n=30\right)$$

(3) $$C_{\mathrm{stock}\;0.2}=1.147\;C_{\mathrm{stock}\;0.3}^{0.996}\left(R^{2}=.998,\;n=30\right)$$

where *S* and *C*_stock_ are the forest area (10^3 ^ha) and FBC stock (Tg C) in a province, respectively, and 0.2 and 0.3 refer to the canopy coverage criterion of the forest stands (>20% and >30%, respectively).

### 2.2 Relative contribution of changes in forest area and FBC density to the accumulation of FBC stock

To explore the relative contribution of changes in forest area and FBC density to the accumulation of FBC stock for each province, we applied the Forest Identity Concept following Kauppi et al. (2006) and Fang et al. (2014):

(4) $$Cc_{\mathrm{stock}}=Cs+Cc_{\mathrm{density}}$$

where *Cc*_stock_, *Cs*, and *Cc*_density_ are the change rate of natural logarithm for FBC stock (*C*_stock_, Tg C), forest area (*S*, 10^3^ ha), and FBC density (*C*_density_, Mg C/ha) over time, respectively.

(5) $$A=Cs/Cc_{\mathrm{stock}}\times 100\%$$

*A* is the relative contribution of the changes in forest area to FBC stock accumulation.

(6) $$D=Cc_{\mathrm{density}}/Cc_{\mathrm{stock}}\times 100\%$$

*D* is the relative contribution of the changes in FBC density to FBC stock accumulation.

### 2.3 Relationship between FBC density and population density

The relationship between the FBC density and population density at the provincial level for each inventory period was analyzed using the least‐squares regression method to fit the following logarithmic model (Wang et al., 2001).

(7) $$C_{d}=a\;\ln\left(P_{d}+b\right)$$

where *C*_*d*_ is the FBC density (Mg C/ha) for each province, *P*_*d*_ is the population density (individuals/km^2^) for each province (Data S3), and *a* and *b* are regression parameters.

(8) $$P_{d}=P_{i}/SP_{i}$$

where *P*_*i*_ is the population for province *i*, which was obtained from the China Compendium of Statistics 1949–2008 (China National Bureau of Statistics, 2010) and the National Bureau of Statistics of the People's Republic of China (National Bureau of Statistics of China, 2014). *SP*_*i*_ is the total area for province *i*, which was acquired from the Atlas of the People's Republic of China (China Mapping Publisher, 1992).

### 2.4 Statistical analysis

Pearson's correlation method was used to assess the correlations between the FBC density and population density for each inventory period. All of the data analyses were performed using PASW Statistics 17.0 for Windows (SPSS Inc. 2009). The standard *P < *0.01 was used as the confidence level for statistical significance. The figures in this paper were completed using SigmaPlot software version 12.5 and ArcGIS 10.2.

### 2.5 Mapping the FBC density distribution

The forest distribution with a spatial resolution of 90 m was extracted from the ecosystem types of China in Ouyang et al. (2016) according to the ecosystem classification system (Ouyang et al., 2015, 2016). The provincial boundary layer with a scale of 1:250,000 was obtained from the National Geomatics Center of China (http://ngcc.sbsm.gov.cn/). We overlapped the forest distribution map with the provincial boundary layer, assigned the provincial FBC density values, and produced the FBC density distribution map.

## 3 RESULTS

### 3.1 Temporal dynamics and spatial distribution of the FBC stock and density

**Figure 1:**
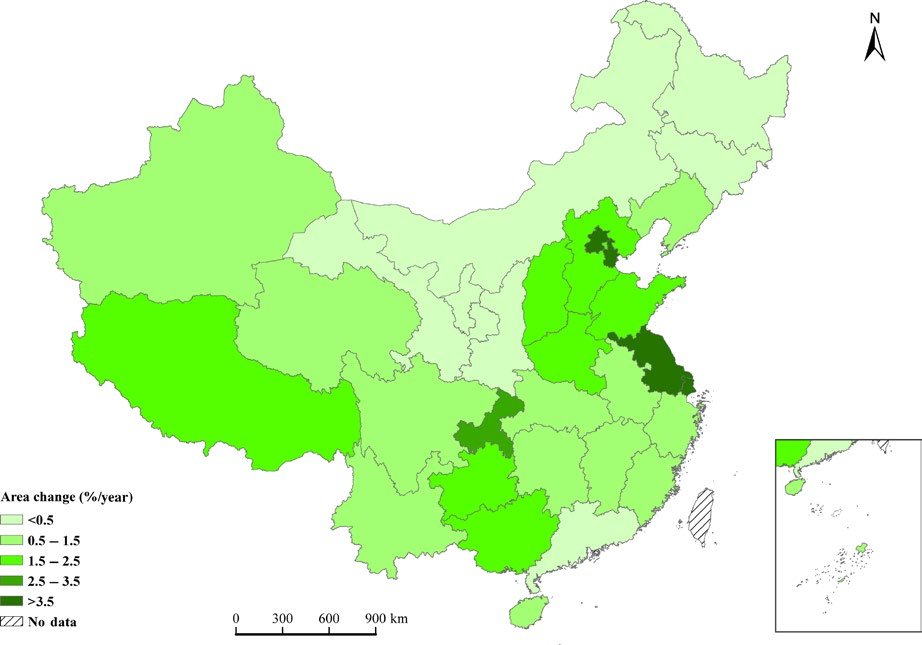
Change of forest area in all provinces, autonomous regions, and municipalities of China from 1977 to 2013

From 1977 to 2013, China's forest area increased by 34.0%, from 122.0 × 10^6^ to 163.5 × 10^6 ^ha (Data S1). And the areal percentage of young and middle‐aged forest stands was large (Table 1), accounting for 69.3% of the total forest area. On the provincial scale, the forest area increased for 87% of provinces, autonomous regions, and municipalities of China, and the forest area in Shanghai increased the most (9.1%/year) (Figure 1).

**Table 1:** Areal percentage and the FBC of young, middle‐aged, and mature forest stands in China from 1977 to 2013

Inventory periods	Percentage (%)			FBC stock (Tg C)			FBC density (Mg C/ha)			Ratio	
	Y	MI	MA	Y	MI	MA	Y	MI	MA	MA/Y	MA/MI
I	35.2	36.6	28.7	607	1,540	2,204	14.1	34.5	62.9	4.5	1.8
II	38.9	32.0	28.5	834	1,369	2,169	16.4	32.7	58.2	3.5	1.8
III	38.2	33.4	29.0	854	1,567	2,625	16.1	33.8	65.3	4.1	1.9
IV	36.8	34.3	28.9	827	1,604	2,587	17.4	36.2	69.4	4.0	1.9
V	33.1	34.8	32.1	874	1,762	3,220	18.5	35.5	70.2	3.8	2.0
VI	33.8	33.4	32.8	992	1,928	3,508	18.9	37.1	68.8	3.7	1.9
VII	32.2	32.3	35.5	1050	2,039	3,976	19.9	38.6	68.6	3.4	1.8
Average	35.5	33.8	30.8	862	1,687	2,899	17.3	35.5	66.2	3.8	1.9

Y, young forest stands; MI, middle‐aged forest stands; MA, mature forest stands, which includes premature, mature, and overmature forests because they were not separated during the 1977–1981 inventory period. I, II, III, IV, V, VI, and VII represent the inventory periods of 1977–1981, 1984–1988, 1989–1993, 1994–1998, 1999–2003, 2004–2008, and 2009–2013, respectively and the absolute value of *a* for the logarithmic curve of the regression model.

**Figure 2:**
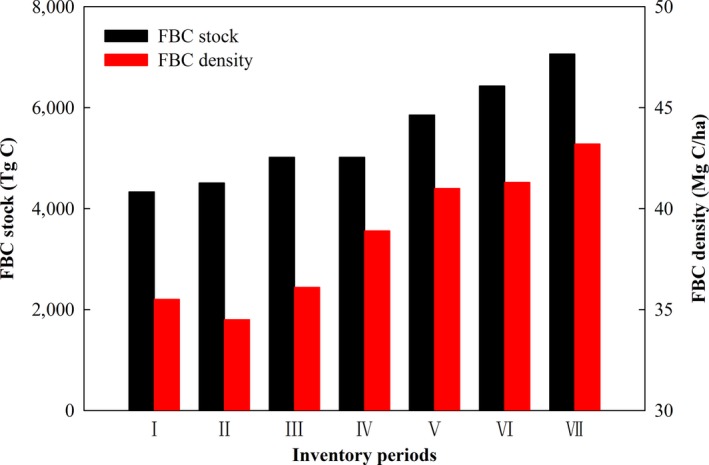
FBC stock and density in China from 1977 to 2013. I, II, III, IV, V, VI, and VII represent the inventory periods from 1977–1981, 1984–1988, 1989–1993, 1994–1998, 1999–2003, 2004–2008, and 2009–2013, respectively

**Figure 3:**
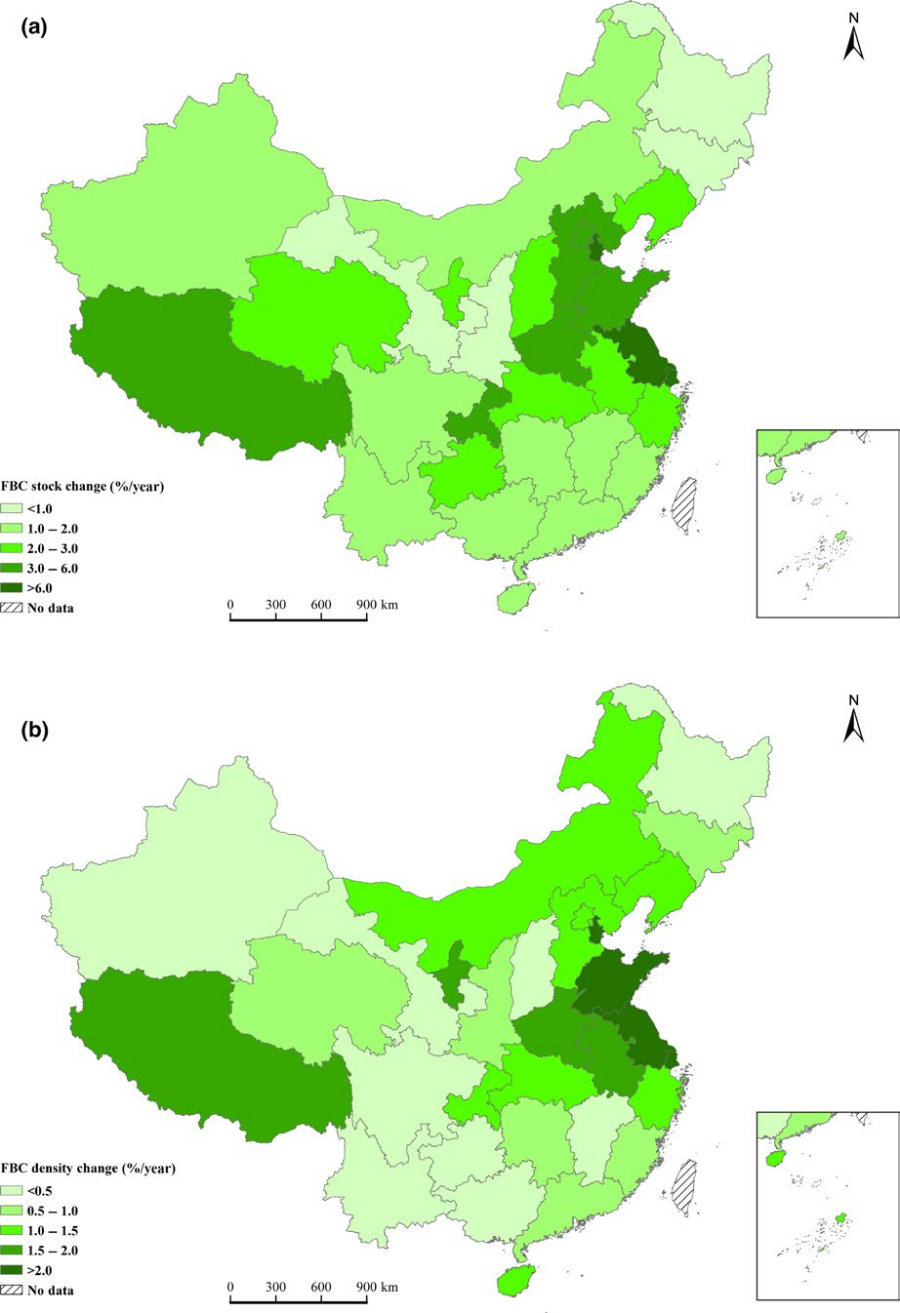
Change in the FBC stock and FBC density in all of the provinces, autonomous regions, and municipalities of China from 1977 to 2013. (a) Mean FBC stock change and (b) mean FBC density change. This figure was created using ArcGIS 10.2. software by Esri. ArcGIS ^®^ and ArcMap are the intellectual property of Esri and are used here under license. For more information on Esri^®^ software, please visit http://www.esri.com/

At the national scale, the FBC stock increased by 62.9%, from 4,335 to 7,064 Tg C; and the FBC density increased by 21.6%, from 35.5 to 43.2 Mg C/ha (Figure 2). The FBC stock and density of young, middle‐aged, and mature also showed increased tendency overall (Table 1). During the study periods, the FBC stock of mature and young forest stands increased by 80.4% and 73.0%, respectively, and the FBC density of young forest stands increased by 40.9%. The FBC stock and density of forest showed increasing trend for all of the provinces, autonomous regions, and municipalities of China (Figure 3a,b). Among these areas, the FBC stock in Shanghai increased the most (12.0%/year), and the FBC density in Shandong Province increased the most (3.4%/year).

**Figure 4:**
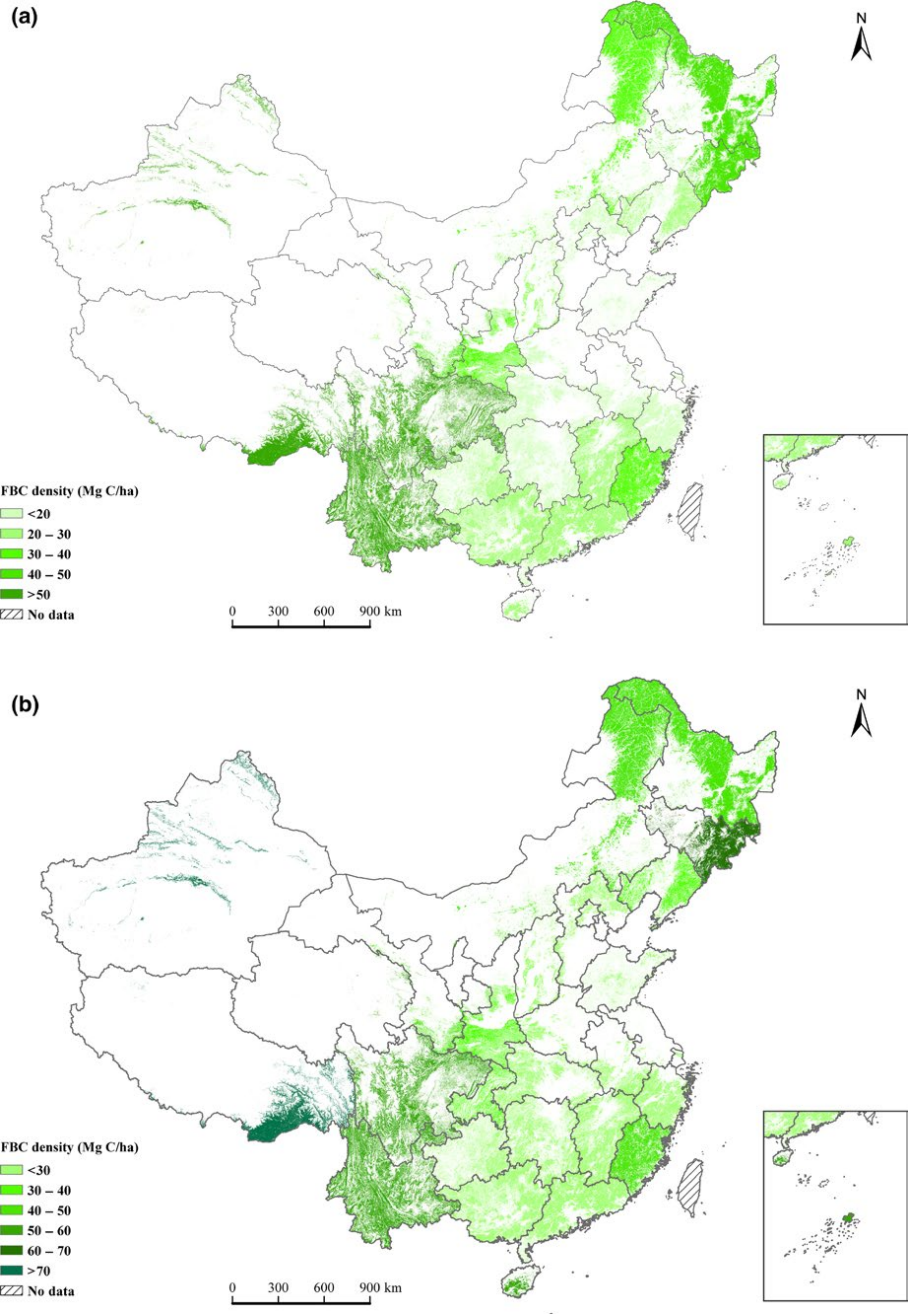
FBC density of the provinces, autonomous regions, and municipalities in China. (a) Mean from 1977 to 1981 and (b) mean from 2009 to 2013. This figure was created using ArcGIS 10.2. software by Esri. ArcGIS ^®^ and ArcMap are the intellectual property of Esri and are used here under license. For more information on Esri^®^ software, please visit http://www.esri.com/

The forest area and the FBC showed considerable spatial variations from 1977 to 2013 over China. Of the national forest area, 38.9%–45.4% were distributed in Heilongjiang, Yunnan, Inner Mongolia, and Sichuan provinces (Data S1). Accordingly, 12.4%–18.4%, 11.3%–12.9%, 10.2%–11.8%, and 10.0%–13.3% of the national FBC stock were located in Heilongjiang, Yunnan, Inner Mongolia, and Sichuan provinces, respectively (Table S1). These four provinces accounted for 44.2%–54.6% of the total FBC stock. The national average FBC densities ranged between 35.5 and 43.2 Mg C/ha, and the provincial average FBC densities ranged between 8.6 and 105.0 Mg C/ha (Figure 4a,b). Based on the estimates from the newest forest inventory, the FBC density increased from the southeastern provinces (22.1–26.1 Mg C/ha in Shanghai, Jiangsu, Zhejiang, and Guangdong) to the northeastern provinces (45.0–62.2 Mg C/ha in Heilongjiang and Jilin provinces) and the western provinces (51.7–105.0 Mg C/ha in Qinghai, Yunnan, Sichuan, Xinjiang, and Tibet) of China (Figure 4b).

### 3.2 Contributions of changes in forest area and FBC density to the FBC stock accumulation

**Figure 5:**
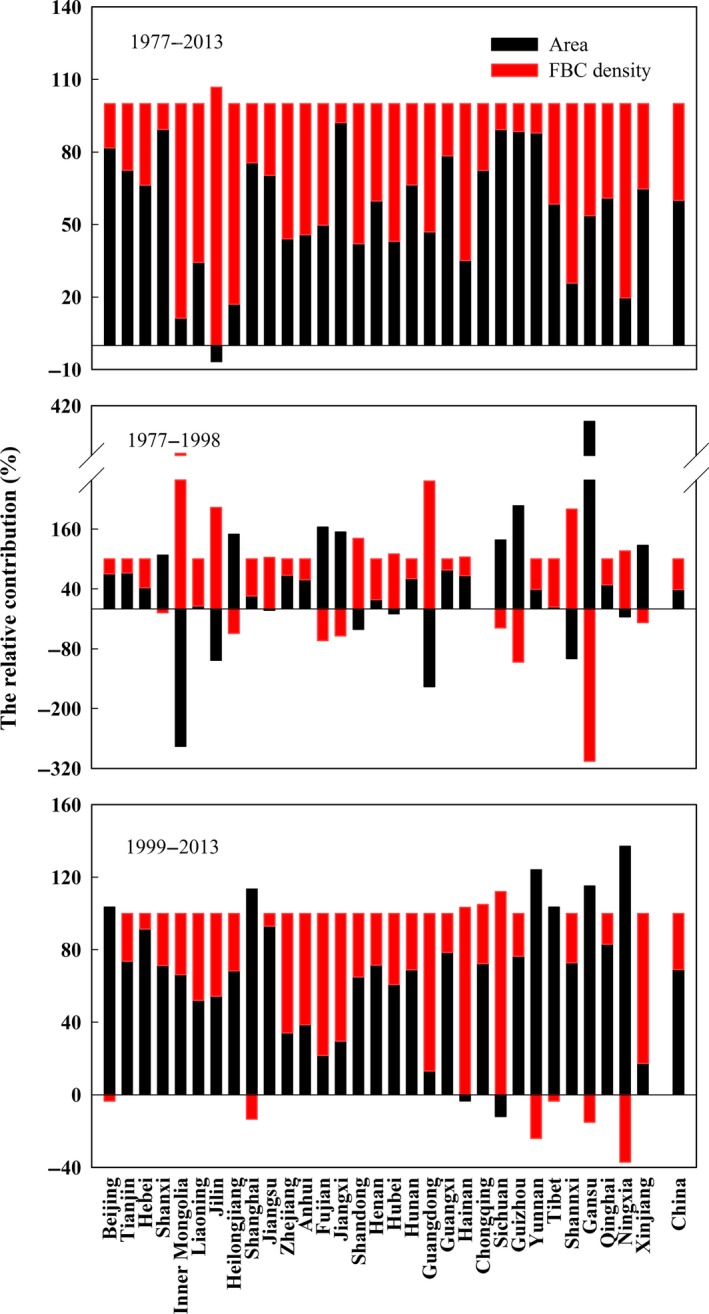
Relative contributions of the forest area and FBC density to the FBC stock accumulation in different provinces, autonomous regions, and municipalities from 1977 to 2013

From 1977 to 2013, the relative contributions of changes in forest area and FBC density to the FBC stock accumulation were 60.0% and 40.0%, respectively (Figure 5). The relative contributions changed over time. Before 1998, the contributions of forest area and FBC density to the FBC stock accumulation were 38.7% and 61.3%, respectively, while they were 69.1% and 30.9% after 1998, respectively. Furthermore, the relative contributions also varied with province. In the provinces where forestation area was large, the increase in FBC stock was mainly induced by forest areal expansion (60.9%–92.1%) during 1977‐2013, while in the others where more natural forests reserved and/or population sparsely distributed, such as Inner Mongolia, Liaoning, Jilin, Heilongjiang, and Hainan, the increase in FBC stock was mainly caused by increasing in the FBC density, with the value from 64.9% to 106.7% at the same periods.

### 3.3 Relationship between the FBC density and population density

**Figure 6:**
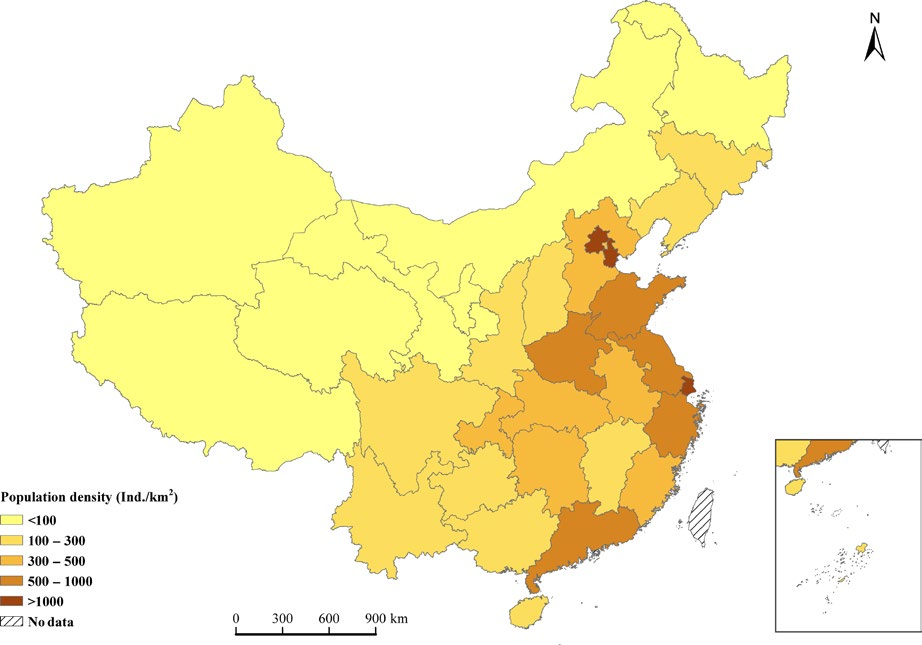
Distribution of the population density for the provinces, autonomous regions, and municipalities in China from 2009 to 2013

At present, the population density increased from the northwestern provinces to the southwestern and northeastern provinces, and then to the eastern and southern provinces (Figure 6).

**Figure 7:**
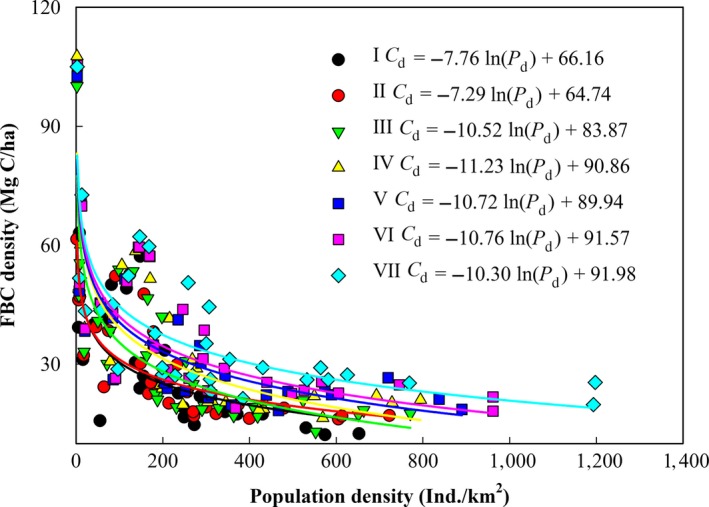
Relationship between the population density and FBC density in China. I: *R* ^2^ = .55, *p *<* *.001, *n* = 28; II:* R* ^2^ = .60, *p *<* *.001, *n* = 29; III:* R* ^2^ = .69, *p *<* *.001, *n* = 29; IV:* R* ^2^ = .70, *p *<* *.001, *n* = 29; V: *R* ^2^ = .67, *p *<* *.001, *n* = 30; VI:* R* ^2^ = .68, *p *<* *.001, *n* = 30; and VII:* R* ^2^ = .65, *p *<* *.001, *n* = 30. I, II, III, IV, V, VI, and VII represent the inventory periods from 1977–1981, 1984–1988, 1989–1993, 1994–1998, 1999–2003, 2004–2008, and 2009–2013, respectively

For each inventory period, a significant negative correlation was observed between the FBC density and population density (*p *<* *.001), which suggests that on a spatial scale, the FBC density decreased gradually with increases in population density (Figure 7). The ln‐transformed relationships between the FBC density and population density indicated that a greater decrease in the FBC density could be induced by increasing the population density when the population density was low, suggesting that the FBC density in sparsely populated regions was more sensitive to population variations compared with the FBC density in more densely populated regions. The absolute value of *a* for the logarithmic curve (i.e., the absolute value of *a* in Equation (7)) increased from 7.76 between 1977 and 1981 to 11.23 between 1994 and 1998, and then it decreased to 10.30 between 2009 and 2013. Thus, the decline in the FBC density with increasing population density peaked from 1994 to 1998, which indicated that the degree of population density‐induced decreases in the FBC density on a spatial scale changed over time.

**Figure 8:**
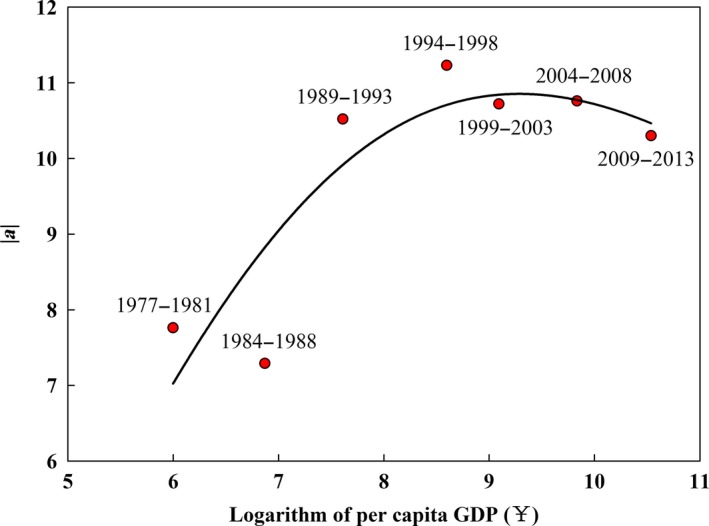
Relationship between economic development and the absolute value of a for the logarithmic curve of the regression model

From 1977 to 1998, the per capita GDP increased from ¥ 403 to ¥ 5419 (China National Bureau of Statistics, 2010), and the absolute value of *a* for the logarithmic curve shown in Figure 7 increased from 7.76 to 11.23 (Figure 8), which indicates that the same degree of increase in the population density led to a greater decrease in FBC density over a spatial scale during 1994–1998 compared with that during 1977–1981. After 1998, the absolute values of *a* for the logarithmic curve decreased from 11.23 to 10.30 and the per capita GDP increased from ¥ 5419 to ¥ 37,703 (China National Bureau of Statistics, 2010; National Bureau of Statistics of China, 2014), indicating that the defined degree of increase in population density was correlated with a lower decline in the FBC density on a spatial scale as the GDP increased.

## 4 DISCUSSION

### 4.1 The influence of forestation on the FBC stock and density

Forestation and forest conservation have been recommended globally as important methods of increasing biomass carbon and mitigating climate change (Sarmiento et al., 2010). The forests of China have been harvested and degraded to support population growth for about 3000 years (Zhang, 2012). However, since the late 1970s, planting trees has become a large‐scale mass activity in China. As a result, the forest area and FBC stock have significantly increased by 34.0% and 62.9% (Data S1; Figure 1), respectively, and the FBC density increased by 7.7 Mg C/ha during the study periods.

The accumulation of the FBC stock was primarily caused by the expansion of forested areas and the regrowth of forest stands (Fang et al., 2014). Approximately 60.0% of the increase in the FBC stock was attributed to the expansion of forested areas, which occurred because of the implementation of a series of forestation projects, such as the Three‐North Shelter Forest Program, Yangtze River Shelter Forest Project, Pearl River Shelter Forest Project, Natural Forest Conservation Program, Grain for Green Program, Fast‐growing Forests in Key Areas Projects, and Beijing and Tianjin Sand Source Control Project (Wang, Innes, Lei, Dai, & Wu, 2007). As a result, forest area increased by 41.5 × 10^6 ^ha during the study period; especially larger‐scale forestation was implemented after 1998, the relative contribution of the increase in forest area to the FBC stock accumulation increased from 38.7% to 69.1% (Figure 5).

### 4.2 The impact of population density on the FBC stock and density

Population density has influenced the spatial heterogeneity of the FBC. In this study, we found that on a spatial scale, the FBC density was significantly negatively correlated with the population density for each forest inventory period in China (*p *<* *.001). The same relationship on the spatial scale has also been found for other major countries or continents for which FBC densities were available (Fig. S1). This relationship occurs because population density is an indicator of human activity that changes the structure and function of forest ecosystems (Josefsson et al., 2009). The FBC densities in the western and northeastern provinces were higher than those in the southeastern provinces of China (Figure 4), which was likely because of lower population density and more natural and mature forests remained in those provinces (Fang, Chen, Peng, Zhao, & Ci, 2001). However, the FBC densities were lower in the southeastern provinces, which are characterized by high population density, planted forests, and young to middle‐aged forests (Fang et al., 2001; Hansen et al., 2013). In China, the biomass carbon densities of mature forest were 3.8 and 1.9 times higher than those of young‐ and middle‐aged forest, respectively (Table 1), and the carbon density of natural forests was 1.66–1.75 times higher than that of planted forests (Fang et al., 2014; Zhang et al., 2013).

Additionally, economic development had a considerable influence on the temporal changes in this relationship, because economic development determines deforestation rates (Ewers, 2006). In our study, there was an inverted U‐shape curve showing the relationship between logarithm of GDP and the slope of the linear regression of FBC density and logarithm of population density (Figure 8). This finding can be explained by the Environmental Kuznets Curve Theory (Ewers, 2006; Kuznets, 1955). During the initial stages of rapid economic development before 1998, economic growth was highly dependent on forests resources, and large forests were harvested to meet the increasing need for wood and land (Naidoo, 2004). This harvest led to the depletion of mature and overmature forest resources, and the deforestation rate reached a maximum of 3.00%/year (Li & Yang, 2000). Consequently, the decline in the FBC density with increase in population density peaked during 1994‐1998. After 1998, incomes and living standard had improved (China National Bureau of Statistics, 2010); thus, forest resources became less crucial to the national economy and public awareness of the natural resources conservation became stronger (Ewers, 2006). During the same year, massive deforestation and erosion contributed to severe flooding along the Yangtze River (Ouyang et al., 2016). These problems eventually prompted the initiation of a series of national key ecological conservation projects at the end of the 20th century, and these programs reduced deforestation and promoted large‐scale forestation. As a result, the defined degree of increase in population density led to a lower decline in FBC density on a spatial scale during 1998‐2013. Therefore, the FBC changes reflected changes in the population density, economic development, forestation, and deforestation. The degree of dependence of the FBC density on the population density has been declining since 1998, which highlights the achievements of China's projects to promote forestation and reduce deforestation, and the importance of human efforts to improve and coordinate human–nature relations, although this improvement occurred after a serious ecosystem degradation.

### 4.3 Implications for forest carbon sequestration

Our results indicated that increases in population density could decrease the FBC density over a spatial scale and the degree of human‐induced decreases in FBC could be reduced via economic development and China's efforts to promote forestation and reduce deforestation. Thus, reduction in population of forested areas could contribute to increase FBC density. In particular, population growth must be controlled and strict protection measures should be implemented in sparsely populated provinces (e.g., Tibet, Yunnan, Heilongjiang, Jilin, and Inner Mongolia) that present large FBC stocks and are more sensitive to population variations. China should vigorously promote urbanization to decrease the influence of the population density on the FBC. In addition, China should continue to support forestation and ecological restoration projects, such as by implementing ecological protections via red‐line planning that designates protection for priority sources of ecosystem services (e.g., carbon sequestration in forest ecosystems) (Ouyang et al., 2016). These actions will increase the positive impacts of human activities on FBC to meet the carbon sink enhancement goals proposed at the 21st session of the United Nations Climate Change Conference of the Parties.

## 5 CONCLUSION

With intensive human disturbances, significant temporal and spatial variations were observed in the FBC. In China, the FBC stock and density increased by 2,729 Tg C and 7.7 Mg C/ha from 1977 to 2013, respectively, which was influenced by human‐driven forestation and ecological restoration efforts. About a half of the FBC stocks were distributed throughout four provinces. The FBC density increased from the densely populated southeastern provinces to the sparsely populated northeastern provinces and western provinces. Furthermore, the regression analysis showed that increases in the population density could significantly decrease FBC density over a spatial scale and indicated that the FBC density was more sensitive to population density variations in sparsely populated provinces where the FBC density was high. These results implied that human activities dominated spatial disturbances of the FBC density and were important for defining the priority protection regions. Simultaneously, the degree of human‐induced decreases in FBC density over a spatial scale has been declining since 1998 because of economic development and China's programs to promote forestation and reduce deforestation. Therefore, the influences of human, including forestation, deforestation, population density, and economic development, play an integral role in determining the temporal and spatial variations of FBC in the anthropogenic era. In addition to developing a deeper understanding of the impacts of humans on the temporal–spatial variations of FBC, changes in FBC under specific human activities should also be investigated.

## CONFLICT OF INTEREST

None declared.
