# Supplementary material for: Human influence on the temporal dynamics and spatial distribution of forest biomass carbon in China
Source: Ecol Evol. 2017 Jul 3;7(16):6220–30. doi: 10.1002/ece3.3188 (PMC5574751; doi:10.1002/ece3.3188)
Supplement: Supplementary file 1 [file ECE3-7-6220-s001.docx]

**Human influence on the temporal dynamics and spatial distribution of forest biomass carbon in China**

Weiwei Liu^1,2,*^, Fei Lu^1,3,*^, Yunjian Luo^4^, Wenjing Bo^1,2^, Lingqiao Kong^1,2^, Lu Zhang^1^, Bojie Liu^1,2^, Zhiyun Ouyang^1^, Xiaoke Wang^1^

^1^State Key Laboratory of Urban and Regional Ecology, Research Center for Eco-Environmental Sciences, Chinese Academy of Sciences, Beijing, China

^2^University of Chinese Academy of Sciences, Beijing, China

^3^Joint Center for Global Change Studies (JCGCS), Beijing, China

^4^School of Horticulture and Plant Protection, Yangzhou University, Yangzhou, Jiangsu, China

**Supporting information**

**Data S1** The forest area of provinces, autonomous regions, and municipalities in China from 1977 to 2013.

| Province | *S* (10^3^ ha) | | | | | | |
| --- | --- | --- | --- | --- | --- | --- | --- |
|  | I | II | III | IV | V | VI | VII |
| Beijing | 86 | 171 | 190 | 207 | 234 | 356 | 407 |
| Tianjin | 11 | 47 | 60 | 43 | 46 | 55 | 73 |
| Hebei | 1496 | 1592 | 1963 | 1900 | 2065 | 2882 | 3097 |
| Shanxi | 917 | 1146 | 1414 | 1471 | 1605 | 1724 | 2030 |
| Inner Mongolia | 16408 | 16548 | 16809 | 13903 | 16082 | 16813 | 17117 |
| Liaoning | 3096 | 3555 | 3482 | 3143 | 3226 | 3613 | 3896 |
| Jilin | 7647 | 7910 | 8058 | 6999 | 7116 | 7267 | 7534 |
| Heilongjiang | 19177 | 19789 | 20493 | 17554 | 17922 | 19126 | 19484 |
| Shanghai | 2 | 3 | 4 | 4 | 6 | 34 | 44 |
| Jiangsu | 221 | 283 | 298 | 217 | 444 | 744 | 1242 |
| Zhejiang | 2980 | 3650 | 3798 | 3448 | 3615 | 3936 | 4101 |
| Anhui | 1939 | 2270 | 2113 | 2339 | 2455 | 2708 | 2917 |
| Fujian | 4581 | 4906 | 5986 | 5499 | 5639 | 5661 | 6067 |
| Jiangxi | 5154 | 5579 | 6456 | 6907 | 7278 | 7681 | 7899 |
| Shandong | 738 | 943 | 830 | 626 | 830 | 1561 | 1603 |
| Henan | 1421 | 1592 | 1688 | 1498 | 1977 | 2834 | 3054 |
| Hubei | 4066 | 4127 | 4270 | 3984 | 4160 | 5078 | 5724 |
| Hunan | 5067 | 4883 | 5348 | 5588 | 6091 | 7265 | 7314 |
| Guangdong | 6123 | 5156 | 6806 | 6788 | 6606 | 6788 | 7148 |
| Guangxi | 5563 | 5459 | 6122 | 6298 | 7475 | 8067 | 9041 |
| Hainan | — | 705 | 783 | 817 | 892 | 842 | 812 |
| Chongqing | — | — | — | — | 1532 | 1820 | 2071 |
| Sichuan | 8217 | 12547 | 13192 | 11977 | 11036 | 11653 | 11789 |
| Guizhou | 2657 | 2514 | 2821 | 3020 | 3443 | 3981 | 4787 |
| Yunnan | 11126 | 10967 | 10979 | 11813 | 13566 | 14727 | 15270 |
| Tibet | 3994 | 3994 | 5077 | 4081 | 8445 | 8411 | 8485 |
| Shannxi | 5330 | 5566 | 5554 | 4926 | 5086 | 5670 | 5743 |
| Gansu | 2232 | 2506 | 2244 | 1922 | 1921 | 2134 | 2451 |
| Qinghai | 247 | 338 | 320 | 305 | 342 | 355 | 379 |
| Ningxia | 112 | 140 | 109 | 102 | 92 | 111 | 129 |
| Xinjiang | 1380 | 1851 | 1609 | 1720 | 1562 | 1692 | 1790 |
| Total | 121985 | 130737 | 138876 | 129094 | 142787 | 155590 | 163497 |

***Notes:*** ‘—’ indicates that no value was available for Hainan and Chongqing in the period I and I, II, III, IV because these two provinces were not separated administratively until 1988 and 1997 and therefore lacked forest area data. I, II, III, IV, V, VI, and VII represent the inventory periods of 1977–1981, 1984–1988, 1989–1993, 1994–1998, 1999–2003, 2004–2008, and 2009–2013, respectively.

**Data S2** The timber volume of provinces, autonomous regions, and municipalities in China from 1977 to 2013.

| Province | *V* (10^4^ m^3^) | | | | | | |
| --- | --- | --- | --- | --- | --- | --- | --- |
|  | I | II | III | IV | V | VI | VII |
| Beijing | 147 | 378 | 446 | 686 | 841 | 1039 | 1425 |
| Tianjin | 19 | 115 | 159 | 160 | 140 | 199 | 374 |
| Hebei | 2650 | 4329 | 5244 | 5948 | 6510 | 8374 | 10775 |
| Shanxi | 3334 | 3791 | 4482 | 5644 | 6199 | 7644 | 9739 |
| Inner Mongolia | 84778 | 86513 | 89676 | 98163 | 110153 | 117721 | 134530 |
| Liaoning | 10039 | 12105 | 13518 | 16137 | 17477 | 20227 | 25046 |
| Jilin | 65697 | 71035 | 75834 | 78657 | 81646 | 84412 | 92257 |
| Heilongjiang | 143663 | 131687 | 134759 | 141069 | 137502 | 152105 | 164487 |
| Shanghai | 2 | 7 | 11 | 24 | 33 | 101 | 186 |
| Jiangsu | 323 | 687 | 812 | 866 | 2285 | 3502 | 6470 |
| Zhejiang | 7918 | 8812 | 9461 | 11122 | 11536 | 17223 | 21680 |
| Anhui | 5458 | 7148 | 6251 | 8296 | 10372 | 13755 | 18075 |
| Fujian | 29638 | 26382 | 32169 | 36491 | 44357 | 48436 | 60796 |
| Jiangxi | 23633 | 16850 | 18089 | 22308 | 32505 | 39530 | 40841 |
| Shandong | 484 | 1056 | 1500 | 1481 | 3202 | 6339 | 8920 |
| Henan | 3189 | 4043 | 4819 | 5259 | 8405 | 12936 | 17095 |
| Hubei | 9860 | 10708 | 11957 | 13224 | 15407 | 20942 | 28653 |
| Hunan | 16021 | 14066 | 15148 | 18459 | 26534 | 34907 | 33099 |
| Guangdong | 20341 | 12760 | 16248 | 19727 | 28366 | 30183 | 35683 |
| Guangxi | 22066 | 20408 | 21359 | 27700 | 36477 | 46875 | 50937 |
| Hainan | — | 5814 | 5696 | 6613 | 7195 | 7274 | 8904 |
| Chongqing | — | — | — | — | 8441 | 11332 | 14652 |
| Sichuan | 104880 | 127301 | 130531 | 144622 | 149543 | 159572 | 168000 |
| Guizhou | 12641 | 10801 | 9391 | 14050 | 17796 | 24008 | 30076 |
| Yunnan | 19073 | 109657 | 110528 | 128347 | 139929 | 155380 | 169309 |
| Tibet | 57778 | 57778 | 123106 | 125337 | 226606 | 224551 | 226207 |
| Shannxi | 25153 | 25881 | 27918 | 30266 | 30776 | 33821 | 39593 |
| Gansu | 16402 | 17209 | 16500 | 17202 | 17504 | 19364 | 21454 |
| Qinghai | 1715 | 2958 | 2960 | 3270 | 3593 | 3916 | 4331 |
| Ningxia | 277 | 542 | 581 | 585 | 393 | 492 | 660 |
| Xinjiang | 20028 | 18326 | 19563 | 25402 | 28040 | 30101 | 33654 |
| Total | 797837 | 809149 | 908717 | 1008564 | 1209764 | 1336259 | 1477909 |

***Notes:*** ‘—’ indicates that no value was available for Hainan and Chongqing in the period I and I, II, III, IV because these two provinces were not separated administratively until 1988 and 1997 and therefore lacked timber volume data. I, II, III, IV, V, VI, and VII represent the inventory periods of 1977–1981, 1984–1988, 1989–1993, 1994–1998, 1999–2003, 2004–2008, and 2009–2013, respectively.

**Data S3** The population density of provinces, autonomous regions, and municipalities in China from 1977 to 2013.

| Province | *P_d_* (ind/km^2^) | | | | | | |
| --- | --- | --- | --- | --- | --- | --- | --- |
|  | I | II | III | IV | V | VI | VII |
| Beijing | 530 | 605 | 651 | 729 | 838 | 962 | 1193 |
| Tianjin | 652 | 723 | 771 | 794 | 890 | 962 | 1198 |
| Hebei | 273 | 300 | 329 | 345 | 358 | 368 | 385 |
| Shanxi | 157 | 174 | 188 | 199 | 210 | 216 | 228 |
| Inner Mongolia | 16 | 17 | 18 | 19 | 20 | 20 | 21 |
| Liaoning | 236 | 256 | 270 | 278 | 288 | 292 | 300 |
| Jilin | 116 | 124 | 131 | 137 | 144 | 145 | 147 |
| Heilongjiang | 70 | 75 | 79 | 82 | 84 | 84 | 84 |
| Shanghai | 1786 | 1957 | 2042 | 2069 | 2681 | 3141 | 3700 |
| Jiangsu | 574 | 613 | 663 | 693 | 720 | 746 | 769 |
| Zhejiang | 372 | 400 | 419 | 432 | 467 | 497 | 533 |
| Anhui | 344 | 374 | 409 | 433 | 439 | 440 | 431 |
| Fujian | 205 | 233 | 253 | 268 | 285 | 295 | 307 |
| Jiangxi | 193 | 214 | 231 | 246 | 252 | 260 | 268 |
| Shandong | 471 | 510 | 552 | 569 | 589 | 605 | 626 |
| Henan | 430 | 479 | 523 | 549 | 574 | 566 | 564 |
| Hubei | 249 | 272 | 295 | 313 | 305 | 307 | 310 |
| Hunan | 247 | 270 | 290 | 303 | 312 | 303 | 311 |
| Guangdong | 286 | 323 | 364 | 422 | 489 | 526 | 581 |
| Guangxi | 147 | 167 | 182 | 194 | 204 | 202 | 199 |
| Hainan | — | 178 | 198 | 216 | 235 | 246 | 258 |
| Chongqing | — | — | — | — | 343 | 342 | 354 |
| Sichuan | 148 | 156 | 165 | 171 | 170 | 169 | 168 |
| Guizhou | 155 | 172 | 188 | 202 | 217 | 211 | 199 |
| Yunnan | 82 | 91 | 98 | 105 | 112 | 117 | 121 |
| Tibet | 1 | 2 | 2 | 2 | 2 | 2 | 2 |
| Shannxi | 137 | 148 | 163 | 172 | 178 | 180 | 182 |
| Gansu | 42 | 46 | 50 | 54 | 56 | 56 | 57 |
| Qinghai | 5 | 6 | 6 | 7 | 7 | 8 | 8 |
| Ningxia | 55 | 64 | 71 | 78 | 85 | 91 | 96 |
| Xinjiang | 8 | 8 | 9 | 10 | 11 | 12 | 13 |
| Total | 101 | 111 | 120 | 126 | 130 | 135 | 139 |

***Notes:*** ‘—’ indicates that no value was available for Hainan and Chongqing in the period I and I, II, III, IV because these two provinces were not separated administratively until 1988 and 1997 and therefore lacked population density data. I, II, III, IV, V, VI, and VII represent the inventory periods of 1977–1981, 1984–1988, 1989–1993, 1994–1998, 1999–2003, 2004–2008, and 2009–2013, respectively.

**Table S1** FBC stock and percentages in all of the provinces, autonomous regions, and municipalities of China from 1977 to 2013.

| Province | FBC stocks (Tg C) | | | | | | | Percentage (%) | | | | | | |
| --- | --- | --- | --- | --- | --- | --- | --- | --- | --- | --- | --- | --- | --- | --- |
|  | I | II | III | IV | V | VI | VII | I | II | III | IV | V | VI | VII |
| Beijing | 1.2 | 2.8 | 3.3 | 4.2 | 5.0 | 6.4 | 8.1 | 0.0 | 0.1 | 0.1 | 0.1 | 0.1 | 0.1 | 0.1 |
| Tianjin | 0.1 | 0.8 | 1.0 | 0.9 | 0.8 | 1.2 | 1.9 | 0.0 | 0.0 | 0.0 | 0.0 | 0.0 | 0.0 | 0.0 |
| Hebei | 22.0 | 30.5 | 37.1 | 38.7 | 40.7 | 54.5 | 66.0 | 0.5 | 0.7 | 0.7 | 0.8 | 0.7 | 0.8 | 0.9 |
| Shanxi | 22.6 | 26.7 | 31.3 | 35.0 | 38.4 | 45.5 | 55.1 | 0.5 | 0.6 | 0.6 | 0.7 | 0.7 | 0.7 | 0.8 |
| Inner Mongolia | 510.6 | 533.0 | 556.6 | 542.2 | 616.2 | 653.9 | 742.2 | 11.8 | 11.8 | 11.1 | 10.8 | 10.5 | 10.2 | 10.5 |
| Liaoning | 70.2 | 81.6 | 86.8 | 90.8 | 97.7 | 113.4 | 137.1 | 1.6 | 1.8 | 1.7 | 1.8 | 1.7 | 1.8 | 1.9 |
| Jilin | 375.5 | 416.3 | 430.6 | 409.1 | 423.4 | 432.6 | 468.4 | 8.7 | 9.2 | 8.6 | 8.2 | 7.2 | 6.7 | 6.6 |
| Heilongjiang | 798.2 | 760.4 | 786.9 | 752.4 | 747.0 | 816.1 | 876.5 | 18.4 | 16.9 | 15.7 | 15.0 | 12.8 | 12.7 | 12.4 |
| Shanghai | 0.0 | 0.0 | 0.1 | 0.1 | 0.2 | 0.6 | 1.0 | 0.0 | 0.0 | 0.0 | 0.0 | 0.0 | 0.0 | 0.0 |
| Jiangsu | 2.7 | 4.8 | 5.5 | 4.8 | 11.8 | 18.4 | 31.3 | 0.1 | 0.1 | 0.1 | 0.1 | 0.2 | 0.3 | 0.4 |
| Zhejiang | 51.8 | 59.5 | 64.1 | 64.3 | 66.2 | 88.7 | 106.9 | 1.2 | 1.3 | 1.3 | 1.3 | 1.1 | 1.4 | 1.5 |
| Anhui | 34.8 | 44.9 | 40.6 | 48.0 | 54.5 | 68.8 | 85.0 | 0.8 | 1.0 | 0.8 | 1.0 | 0.9 | 1.1 | 1.2 |
| Fujian | 153.2 | 146.3 | 184.2 | 171.3 | 195.5 | 218.3 | 269.6 | 3.5 | 3.2 | 3.7 | 3.4 | 3.3 | 3.4 | 3.8 |
| Jiangxi | 135.0 | 112.3 | 123.6 | 136.4 | 169.0 | 203.2 | 214.7 | 3.1 | 2.5 | 2.5 | 2.7 | 2.9 | 3.2 | 3.0 |
| Shandong | 6.9 | 9.2 | 10.7 | 10.3 | 18.5 | 35.4 | 43.7 | 0.2 | 0.2 | 0.2 | 0.2 | 0.3 | 0.6 | 0.6 |
| Henan | 24.7 | 30.1 | 35.2 | 32.8 | 49.0 | 72.3 | 88.9 | 0.6 | 0.7 | 0.7 | 0.7 | 0.8 | 1.1 | 1.3 |
| Hubei | 67.7 | 69.9 | 83.0 | 82.5 | 89.6 | 115.9 | 149.9 | 1.6 | 1.5 | 1.7 | 1.6 | 1.5 | 1.8 | 2.1 |
| Hunan | 97.0 | 87.7 | 99.4 | 114.1 | 135.6 | 169.1 | 168.6 | 2.2 | 1.9 | 2.0 | 2.3 | 2.3 | 2.6 | 2.4 |
| Guangdong | 134.1 | 90.2 | 115.8 | 125.6 | 152.6 | 159.1 | 186.6 | 3.1 | 2.0 | 2.3 | 2.5 | 2.6 | 2.5 | 2.6 |
| Guangxi | 132.9 | 123.2 | 138.5 | 155.9 | 189.3 | 226.3 | 247.1 | 3.1 | 2.7 | 2.8 | 3.1 | 3.2 | 3.5 | 3.5 |
| Hainan | — | 26.9 | 32.9 | 33.9 | 36.7 | 36.9 | 41.0 | — | 0.6 | 0.7 | 0.7 | 0.6 | 0.6 | 0.6 |
| Chongqing | — | — | — | — | 41.4 | 52.6 | 64.6 | — | — | — | — | 0.7 | 0.8 | 0.9 |
| Sichuan | 469.6 | 598.0 | 614.2 | 616.7 | 630.2 | 667.0 | 703.9 | 10.8 | 13.3 | 12.2 | 12.3 | 10.8 | 10.4 | 10.0 |
| Guizhou | 71.6 | 63.7 | 59.2 | 76.2 | 90.0 | 113.7 | 139.4 | 1.7 | 1.4 | 1.2 | 1.5 | 1.5 | 1.8 | 2.0 |
| Yunnan | 556.6 | 572.7 | 590.9 | 648.9 | 698.7 | 748.4 | 798.1 | 12.8 | 12.7 | 11.8 | 12.9 | 11.9 | 11.6 | 11.3 |
| Tibet | 245.8 | 245.8 | 508.3 | 439.1 | 866.2 | 885.7 | 890.8 | 5.7 | 5.4 | 10.1 | 8.8 | 14.8 | 13.8 | 12.6 |
| Shannxi | 162.2 | 167.9 | 179.1 | 175.6 | 177.1 | 192.9 | 216.8 | 3.7 | 3.7 | 3.6 | 3.5 | 3.0 | 3.0 | 3.1 |
| Gansu | 89.4 | 98.6 | 91.7 | 86.2 | 87.2 | 96.2 | 106.5 | 2.1 | 2.2 | 1.8 | 1.7 | 1.5 | 1.5 | 1.5 |
| Qinghai | 9.7 | 15.6 | 15.2 | 15.1 | 16.6 | 17.9 | 19.6 | 0.2 | 0.3 | 0.3 | 0.3 | 0.3 | 0.3 | 0.3 |
| Ningxia | 1.8 | 3.4 | 3.3 | 3.1 | 2.4 | 2.9 | 3.7 | 0.0 | 0.1 | 0.1 | 0.1 | 0.0 | 0.0 | 0.1 |
| Xinjiang | 87.1 | 87.0 | 89.2 | 103.5 | 110.1 | 118.2 | 130.1 | 2.0 | 1.9 | 1.8 | 2.1 | 1.9 | 1.8 | 1.8 |
| Total | 4335 | 4510 | 5018 | 5018 | 5857 | 6430 | 7064 | 100 | 100 | 100 | 100 | 100 | 100 | 100 |

***Notes:*** ‘—’ indicates that no value was available for Hainan and Chongqing for the 1977–1981 and 1977–1998 periods because these two provinces were not separated administratively until 1988 and 1997, respectively, and therefore lacked inventory data. I, II, III, IV, V, VI, and VII represent the inventory periods of 1977–1981, 1984–1988, 1989–1993, 1994–1998, 1999–2003, 2004–2008, and 2009–2013, respectively





**Figure S1** Relationship between the FBC density and population density in different countries or regions of the world ([FAO, 2010](#_ENREF_12); [Pan et al., 2011](#_ENREF_43)).

**References**

FAO [Food and Agriculture Organization of the United Nations]. (2010). Global forest resources assessment 2010: main reports (pp. 218-228, 273-276). Rome (Italy).

Pan, Y., Birdsey, R. A., Fang, J., Houghton, R., Kauppi, P. E., Kurz, W. A., Phillips, O. L., Shvidenko, A., Lewis, S. L., Canadell, J. G., Ciais, P., Jackson, R. B., Pacala, S. W., McGuire, A. D., Piao, S., Rautiainen, A., Sitch, S., & Hayes, D. (2011). A large and persistent carbon sink in the world's forests. *Science*, *333*, 988-993.
